# Pulmonary Atypical Adenomatous Hyperplasia: Diagnostic and Therapeutic Implications

**DOI:** 10.7759/cureus.6079

**Published:** 2019-11-05

**Authors:** Cam Nguyen, Nicholas K Larsen, Nick Dietz, Gopi Sirineni, Marcus Balters

**Affiliations:** 1 Radiation Oncology, Creighton University School of Medicine, Omaha, USA; 2 Surgery, Creighton University School of Medicine, Omaha, USA; 3 Pathology, Creighton University School of Medicine, Omaha, USA; 4 Radiology, University of Alabama, Birmingham, USA

**Keywords:** atypical adenomatous hyperplasia, pulmonary aah, adenocarcinoma, bronchioloalveolar carcinoma, stereotactic body radiotherapy

## Abstract

Lung cancer still remains one of the most common cancers throughout the world, especially in smokers. Adenocarcinoma is now the predominant histological type in many western countries. The etiology of adenocarcinoma is unknown, but evidence suggests that atypical adenomatous hyperplasia (AAH) may act as a precursor lesion. Here we present two case reports of patients diagnosed with AAH on biopsy, highlighting 1) available treatment strategies and 2) AAH’s progression to adenocarcinoma. A review of AAH is warranted as little literature is currently available regarding its treatment strategies, especially in light of its role as a precursor to adenocarcinoma.

In this review, we will address the following topics:

1. What is the pathophysiology of AAH? 2. What is the natural history of AAH and its risk of malignant transformation? 3. When is surgery recommended? 4. What is the role of stereotactic body radiotherapy (SBRT) in the rare patient who refuses surgery?

## Introduction

Lung cancer is one of the most common malignancies in the world, especially in smokers. Pulmonary adenocarcinoma is the most common cell type, though the exact etiology of adenocarcinoma is unknown. Pulmonary atypical adenomatous hyperplasia (AAH) may act as a precursor lesion to adenocarcinoma [1]. Some pathologists think pulmonary AAH is similar to breast ductal carcinoma in situ, which is a precursor lesion of invasive breast carcinoma. Most adenocarcinomas are located at the periphery of the lung, making them difficult to detect by means of flexible fiberoptic endoscopy. Thus, these lesions are often diagnosed with CT-guided trans-thoracic needle biopsy.

A number of different terms have been used in the literature to refer to AAH. These include but are not limited to the following: atypical alveolar epithelial hyperplasia [2], atypical bronchioloalveolar cell hyperplasia [3,4], alveolar atypical hyperplasia [5], and bronchioloalveolar cell adenoma [6]. AAH is best viewed as a small proliferative lesion comprising single-layered atypical cells that appear similar to Clara cells or type II alveolar cells lining along the alveolar septa. Cells seen in AAH show various degrees of atypia, such as nuclear enlargement, hyperchromasia, pleomorphism, prominent nucleoli, variable cellularity, and general architectural disarray.

Some studies have suggested that the grade of atypia in AAH is less than that of adenocarcinoma. However, the definitive histologic criteria for diagnosis of AAH remain to be defined. In fact, the distinction between AAH with severe atypia and well-differentiated bronchioloalveolar carcinoma (BAC) can be very difficult on the basis of histopathology alone. The diagnosis is frequently made using a combination of radiographic and clinical findings. The natural history of AAH is poorly understood. Many clinicians regard AAH as a precursor lesion of BAC [4,7], while others regarding it as an adenoma, or conversely, as an extremely well differentiated BAC [3,6,8].

Here, we present two patients' cases that 1) demonstrate the different options for AAH treatment and 2) demonstrate AAH acting as a precursor lesion to adenocarcinoma. In addition, this manuscript aims to discuss the epidemiology and recent advances in the understanding of AAH, which may help diagnostic pathologists recognize AAH. This review further discusses studies that have used various ancillary methodologies such as morphometry, immunohistochemistry, cytogenetic methods, and molecular analyses.

## Case presentation

**Figure 1:**
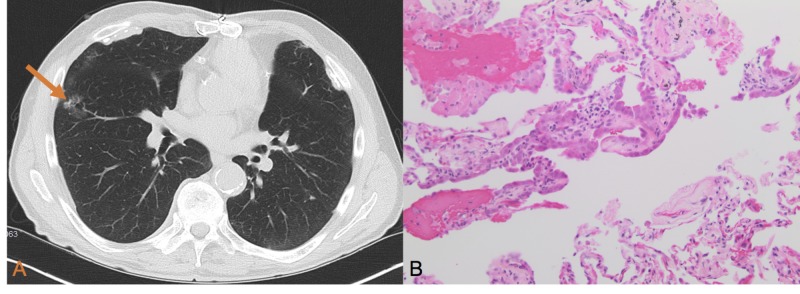
(A) CT scan showing a 2.8-cm ground glass opacity nodule in the right middle lobe (orange arrow). There was no evidence of mediastinal adenopathy. (B) The lung parenchyma shows a sharply demarcated area of alveolar space lined by enlarged cells with prominent nucleoli and mild nuclear pleomorphism (H&E, 200x magnification).

A 65-year-old man with a 60-pack-year smoking history presented with a right middle lobe ground glass opaque (GGO) nodule which measured 1.9 cm. One year later, the nodule progressed in size to 2.8 cm (Figure 1A). CT scan showed there was no mediastinal adenopathy present in the chest. Transthoracic needle aspiration was performed and demonstrated atypical adenomatous hyperplasia (AAH) (Figure 1B). The patient was recommended to undergo right middle lobectomy, but refused and was referred for a radiation oncology consultation. The case was presented at the multidisciplinary tumor conference where there were multiple differing opinions on the future management considerations for this patient. To date, there is no evidence supporting stereotactic body radiotherapy (SBRT) use for AAH. Observation was recommended with a serial CT scan every three months. At the two-year follow-up visit with repeat chest CT, the GGO is unchanged in appearance or size.

**Figure 2:**
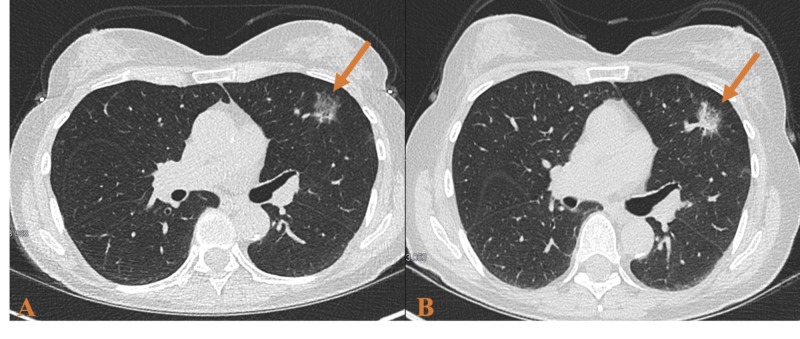
(A) CT scan showing a 2.3 x 1.3 x 0.9 cm ground glass opacity nodule in the anterior left upper lobe. (B) Follow-up CT scan showed the solid nodular component increasing to 2.8 x 1.4 x 1.0 cm.

**Figure 3:**
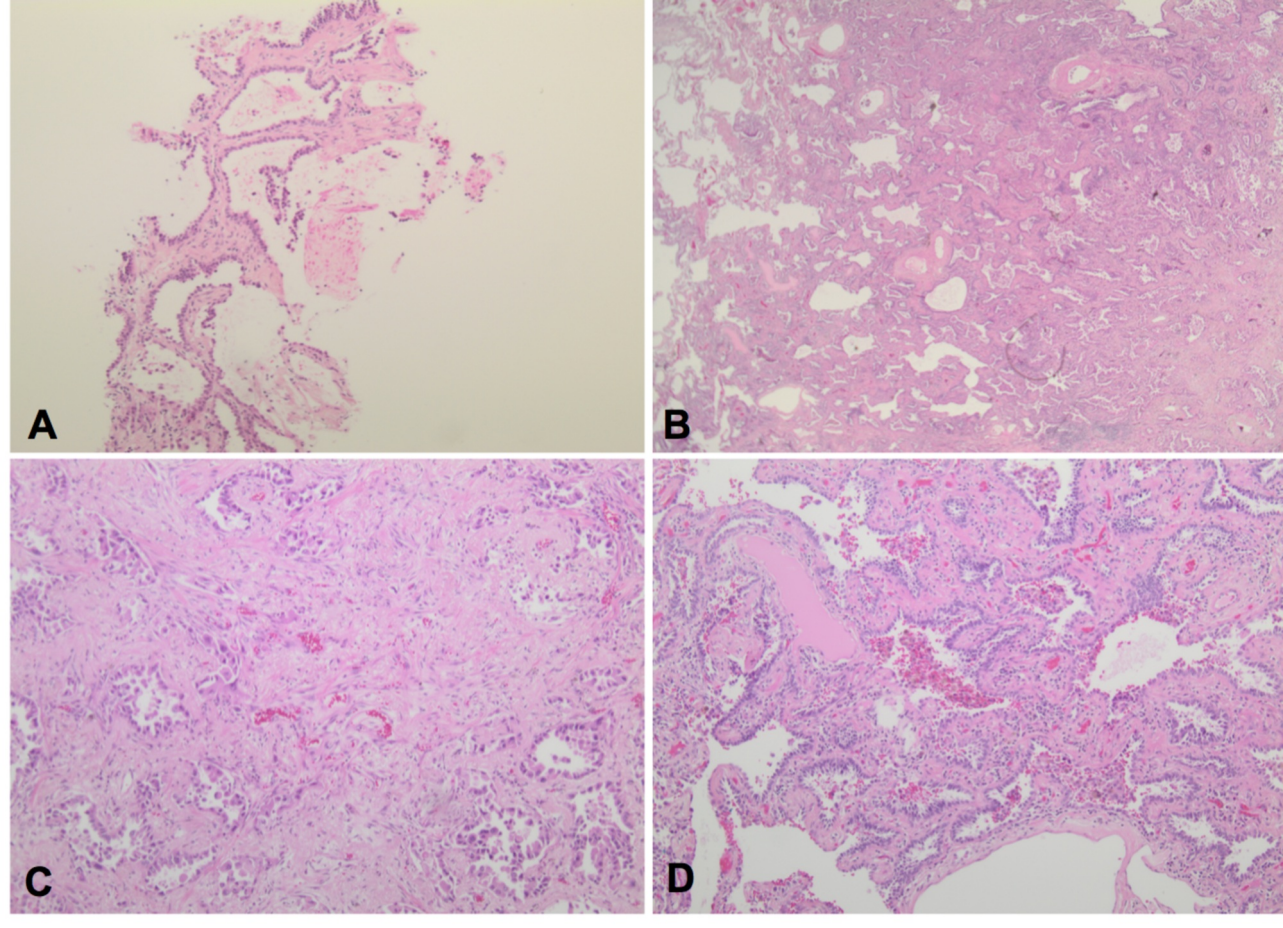
(A) Mildly atypical cells continuously line alveolar spaces without evidence of invasion or pleomorphism consistent with atypical adenomatous hyperplasia (AAH) (H&E, 100x magnification). (B) Mass lesion showing peripheral lepidic areas with preserved architecture and a central area of invasive carcinoma (H&E, 20x magnification). (C) Central area showing invasive carcinoma (acinar pattern) with a desmoplastic stroma (H&E, 100x magnification). (D) Peripheral area of tumor showing preserved alveoli lined by atypical cells (lepidic pattern) (H&E, 100x magnification).

In another case, a 73-year-old female with a long smoking history was referred to thoracic surgery for a slow-growing nodule in her left upper lobe. A CT scan showed a 2.3 x 1.3 x 0.9 cm GGO nodule in the anterior left upper lobe (Figure 2A). At this time, a three-month follow-up was recommended. A repeat scan showed unchanged size and configuration of the nodule. The patient was recommended to follow-up in six months, but she did not return for almost a year. At that time, a biopsy was performed showing alveolar atypia consistent with AAH (Figure 3A). A repeat CT was performed six months following the biopsy and the nodule became "semisolid" with both solid and GGO features here (Figure 2B). At no time did any of the CT scans demonstrate mediastinal adenopathy or other abnormality. The patient's case was presented at the multi-disciplinary tumor board and surgical consultation was recommended.

Upon presentation to thoracic surgery, the patient continued to smoke. She denied any symptoms of cough, weight loss, or change in appetite. Given the location and concerns for potential malignancy within the previously identified atypical adenomatous hyperplasia, the patient was consented for lobectomy. The patient underwent an uneventful left upper lobectomy and lymph node dissection. Final pathology showed two foci of invasive moderately differentiated adenocarcinoma, acinar pattern (Figure 3B-3D). Multiple lymph nodes were positive for malignancy including 4L, 11L and other peribronchial nodes with no evidence of distant metastases or brain metastases on CT and PET scan. The patient’s final pathology demonstrated her to be T3N2M0. She was referred to medical oncology for chemotherapy and radiation oncology for radiation.

Together these cases demonstrate the varying opinions on clinical management of AAH and to date, there is no empiric treatment for AAH. Traditional treatment options include surgical resection or observation with serial CT scans. The role of SBRT is unknown due to lack of data on the use of SBRT in treating AAH. In addition, the latter case demonstrates that AAH can act as precursor lesion of adenocarcinoma.

## Discussion

Adenocarcinoma is now the predominant histologic type of lung cancer in many western countries. Clearly, an understanding of possible precursor lesions for this increasingly frequent lung cancer would help establish a diagnostic classification, as well as guide management strategies. Many investigators have suggested that AAH is a precursor lesion of adenocarcinoma [1]. The World Health Organization (WHO) currently acknowledges AAH as a pre-invasive lesion, but there can be difficulty trying to distinguish AAH from the non-mucinous variant of BAC [9]. The WHO defines AAH as a "focal lesion, often 5 mm or less in diameter, in which the involved alveoli and respiratory bronchioles are lined by monotonous, slightly atypical cuboidal to low columnar epithelial cells with dense nuclear chromatin, prominent nucleoli and scant cytoplasm" [9].

Epidemiology and pathology

Population-based epidemiologic studies addressing the frequency of AAH have not been reported. However, there are some studies that provide some data on the frequency of AAH in hospital-based populations. Miller examined 247 cases of surgically resected lung carcinomas by sectioning all areas of the lung transversely at 1.0- to 1.5-cm intervals [6]. They found 23 (9.3%) of lesions were bronchioloalveolar cell adenomas, otherwise known as AAH. Other authors have reported a frequency of 5 to 20% of AAH in surgically resected lungs for pulmonary carcinoma [2,10-13]. Weng et al. reviewed the literature that examined the incidence of AAH and found that it was especially higher in specimens obtained from patients with primary lung carcinoma (20.0%) than in those with non-primary lung carcinoma (4.8%) [10]. Among many histopathologic types of lung carcinoma, AAH has been found most frequently in resected lungs of patients with pulmonary adenocarcinoma, especially well differentiated papillary adenocarcinoma and BAC [2,10-13]. Other authors reported the incidences of AAH in the range of 1.2 to 12% in surgically resected adenocarcinoma of the lung [5,8,12,14]. The actual incidence of AAH in the general population is unknown. Chapman and Kerr have recently demonstrated that a greater percentage of women with adenocarcinoma, when compared to men, had AAH (30.2% vs. 18.8%) [13].

Other means to assess AAH have been used, such as morphometry, immunohistochemistry, and molecular studies; though, some morphometric, molecular analyses and immunohistochemical studies are not always available to many practicing pathologists. Therefore, diagnosis of AAH will likely continue to rest on subjective histologic criteria. Some authors suggested the following guidelines for the diagnosis of AAH: prominent cuboidal to low columnar alveolar epithelial cells with different degrees of atypia, less than that seen in the more common adenocarcinoma; irregularly bordered focal proliferations of atypical cells spreading along the pre-existing alveoli; increased cell size and nuclear-cytoplasmic ratio with hyperchromasia and prominent nucleoli; intercellular attachment of atypical cells generally intact with occasional existence of empty-looking spaces between them without high cellularity.

On the other hand, any precursor lesion of a cancer should fulfill certain criteria [15]. First, it should exist before the development of the malignancy. Second, the precursor lesion should demonstrate cellular atypia and/or molecular changes and is consistent with progressive cellular transformation. Third, the precursor lesion should be more common than the malignancy, in other words, it should not occur so rarely that transformation to neoplasia is rare.

Guidelines for screening, follow-up, and intervention

The current recommendation of low-dose CT screening for patients at risk will undoubtedly pick up more pulmonary lesions; some of these lesions eventually will be AAH. Current guidelines for lung cancer screening from the US Preventative Services Task Force recommend annual screening with low-dose CT scan for adults 55-80 years of age who have at least a 30-pack year smoking history. The patient must be a current smoker or have quit within the last 15 years. Screening ends if the patient has not smoked for 15 years, develops a life limiting illness, or becomes unable (or unwilling) to undergo curative surgery [16]. However, medically inoperable patients are often treated with SBRT, and this led to modifications of current guidelines for lung cancer screening.

AAH is nearly indistinguishable from focal inflammation and BAC, with each appearing as ground glass opacities (GGO) [17]. Regarding pure GGO, current Fleischner Guidelines recommend no follow-up if smaller than 6 mm in diameter. For those pure GGO 6 mm or larger, follow-up scanning is recommended at 6-12 months and then every two years thereafter until five years [18]. In those nodules that are less than 6 mm, follow-up can be considered at two and four years if the nodule appears suspicious [18]. The various etiologies of GGO create a dilemma for clinicians, who need to make decisions on further monitoring, versus biopsy and/or resection.

Management of pulmonary AAH varies, with a conservative approach recommended by Ritter [19]. This conservative approach stated that small lesions found incidentally, without a gross correlate, that lack convincing pathologic features of malignancy, can be safely designated as AAH. In contrast, lesions that represent clinically or grossly observed lesions with compelling atypical features can be designated as malignant. A multidisciplinary approach to these lesions involving surgeons, oncologists, radiation oncologists, radiologists, and pathologists is warranted to identify worrisome clinical, pathologic, or radiologic features which may necessitate a more aggressive approach.

The diagnostic guidelines for AAH for the pathologist can be controversial. Similarly, managing this condition once diagnosed is also difficult, and there are no definitive guidelines. Previous authors have discussed treatment options ranging from conservative observation to surgical resection. Some studies show observation is safe with compliant patients who show up for routine CT surveillance at appropriate frequency (usually every six months for the AAH lesions). The surgical series showed that AAH lesions were removed with a variety of procedures ranging from wedge resection, segmentectomy, to lobectomy [20].

Currently, there is very little data supporting the use of stereotactic body radiation therapy (SBRT) for pulmonary AAH. On the rare occasion of patients with pulmonary AAH, which carries a 10-15% chance of harboring malignancy, SBRT may be justified in the non-compliant patients that may be lost to follow-up.

## Conclusions

Is AAH a pre-malignant lesion? The current data suggests that AAH plays a role in the development of pulmonary adenocarcinoma, particularly in the development of BAC. Further work is needed to establish widely acceptable histopathologic criteria for the diagnosis of AAH. In addition, further study is warranted on the role of SBRT for patients with AAH who deny surgical intervention. The intriguing nature of pulmonary AAH in the development of pulmonary adenocarcinoma deserves further study.
